# Potential Application of Copper Aspirinate in Preventing and Treating Thromboembolic Diseases

**DOI:** 10.1155/MBD.1998.123

**Published:** 1998

**Authors:** Weiping Liu, Huizhou Xiong, Yikun Yang, Ling Li, Zhiqiang Shen, Zhihe Chen

**Affiliations:** 1 Institute of Precious Metals Kunming 650221 China; 2 Kunming Medical College Kunming 650031 China

## Abstract

The efficacy of copper aspirinate against thrombotic diseases has been tested in animal models. The results show that copper aspirinate, following ig pretreatment for 7 days at 0.012mmol/kg markedly prolonged the bleeding time and inhibited the mortality induced by arachidonic acid (AA) in mice. On cereral ischemia model pretreatment with 0.018mmol/kg copper aspirinate ig significantly increased survival of animals and the density of intact hippocampal CA1 cells and decreased brain calcium concentration. Its anticerebral ischemia activity was superior to or equal to nimodipine. It is, therefore, suggested that copper aspirinate is very promising in becoming an antithrombotic drug in preventing and treating thrombotic diseases.


### POTENTIAL APPLICATION OF COPPER ASPIRINATE IN PREVENTING AND TREATING THROMBOEMBOLIC DISEASES

Weiping Liu*[1], Huizhou Xiong[1], Yikun Yang[1], Ling Li[2], Zhiqiang Shen[2], and Zhihe Chen[2]

[1] Institute of Precious Metals, Kunming 650221,China
[2] Kunming Medical College, Kunming 650031,China

**Abstract**

The efficacy of copper aspirinate against thrombotic diseases has been tested in animal models. The results show that copper aspirinate following ig pretreatment for 7 days at 0.012mmol/kg markedly prolonged the bleeding time and inhibited the mortality induced by arachidonic acid (AA) in mice. On cereral ischemia model, pretreatment with 0.018mmol/kg copper aspirinate ig significantly increased survival of animals and the density of intact hippocampal CA1 cells,and decreased brain calcium concentration. Its anticerebral ischemia activity was superior to or equal to nimodipine. It is,therefore,suggested that copper aspirinate is very promising in becoming an antithrombotic drug in preventing and treating thrombotic diseases.

### 1. Introduction

Copper aspirinate is a copper complex of aspirin (see Fig. 1) and has been demonstrated to be more active as an antiinflammatory agent and less irritant to the digestive tract than aspirin [1][2]. Moreover it has,contrary to aspirin,antiulcer activity [3][4]. Consequently it is recommended that copper aspirinate should be clinically used to treat arthritic and other degenerative diseases. Recently we discovered that it possesses much greater efficacy than aspirin against arachidonic acid (AA) induced platelet aggregation [5],which suggests that copper aspirinate may be a potential antithrombotic drug. In order to explore its usage in the prevention and therapy of thromboembolic disorders related to undue aggregation of platelets, we have examined its antithrombotic and anticerebral ischemia activities in animal models,and report our results here.

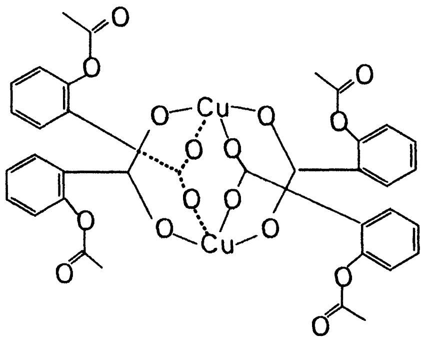

Fig.1 The chemical structure of copper aspirinate

### 2. Experimental

Copper aspirinate was synthesized by a published method [3] and an aqueous suspension in 5% propylene glycol and 1.4% polyvinyl alcohol was prepared just before administration. Groups of 20 to 25g ICR mice and 50 to 70g Mongolian gerbils of either sex were used as experimental animals,and aspirin or nimodipine was selected for comparison with copper aspirinate. Data obtained from the experiments were analyzed by the t−test.

***Effect of copper aspirinate on the tail bleeding time***

Groups of 10 ICR mice were given copper aspirinate, aspirin or the vehicle intragastrically (ig) for 7 days,and then the tail bleeding time of each group was measured using Wang's method [6]. Results are given in Table 1.

***Effect of copper aspirinate on mortality caused by thrombosis***

Groups of 30 IRC mice,after administrated with copper aspirinate or aspirin or vehicle ig for 7 days, were injected with 80mg/kg AA in the tail vein to induce thrombosis [7],the number of mice dying in each group was recorded and the mortality calculated (Ta[illegible] 2).

***Effect of copper as[illegible]nate on cerebral ischemia injury***

Groups of gerils were treated with copper aspirinate or vehicle by the ig route or with nimodipine by the ip (intraperitoneal) route for 7 days just before cerebral ischemia injury was produced by occlusion of the bilateral carotid arteries followed by reperfusion. Ischemia caused by occlusion is similar to that caused by thrombosis in man. For gerbils following 20 min occlusion and one day of reperfusion,the death number of each group within 24 hours was determined,and parietotemporal cortex was dissected out for the determination of calcium and water content by means of the documented method in the literature [8]. For gerbils following 10 min occlusion and 7 day reperfusion,the density of neurons in the hippocamus CA1 sector,in terms of the number of intact neurons in two fields vision,was determined under a photomicroscope according to a known method [9]. The results were presented in Tables 3,4 and 5.

Table 1. Effect of 0.012mmol/kg copper aspirinate and 0.056mmol/kg aspirin ig on tail bleeding time, $\bar{x} \pm s$, $n=10$, $^{*}P<0.05$ vs control

| Group | Bleed time (min) |
|---|---|
| Control | 11.6 ± 1.7 |
| Aspirin | 13.3 ± 3.6 |
| Copper aspirinate | 17.8 ± 3.7* |

Table 2 Effect of 0.012mmol/kg copper aspirinate and 0.056mmol/kg aspirin ig on the mortality caused by AA, $\bar{x} \pm s$, $n=30$, $^{**}P<0.01$ vs control

| Group | Mortality |
|---|---|
| Control | 87% |
| Aspirin | 27%** |
| Copper aspirinate | 13%** |

Table 3. Effect of 0.018mmol/kg ig copper aspirinate and 0.024mmol/kg ip nimodipine on the mortality of gerbils after ischemia, *$P<0.05$ vs control

| Group | number of animals | Mortality |
|---|---|---|
| Control | 15 | 33% |
| Nimodipine | 18 | 6%* |
| Copper aspirinate | 14 | 7%* |

Table 4 Effect of 0.018mmol/kg ig copper aspirinate and 0.024mmol/kg ip nimodipine on brain water and calcium content in gerbils following ischemia, $x \pm s$, $n=7$, **$P<0.01$ vs control

| Group | $H_2O$(%) | $Ca^{2+}$($\mu$mol/g wet wt) |
|---|---|---|
| Control | 75.5 ± 2.08 | 4.2 ± 0.88 |
| Nimodipine | 69.8 ± 2.08 | 1.9 ± 0.60** |
| Copper aspirinate | 75.8 ± 0.68 | 1.3 ± 0.69** |

Table 5. Number of hippocampal CA1 cells remaining in cerebal ischmia gerbils pretreated with 0.018mmol/kg ig copper aspirinate or 0.024mmol/kg ip nimodipine, $x \pm s$, $n=6$, **$P<0.01$ vs control

| Group | Number of cell |
|---|---|
| Control | 38.3 ± 14.3 |
| Nimodipine | 63.2 ± 14.9** |
| Copper aspirinate | 76.6 ± 10.8** |

**3. Results and Disscussion**

Effects of copper aspirinate on the tail bleeding time and on the mice mortality caused by AA are listed,respectively,in Table 1 and 2. These data show that ig administration of 0.012mmol/kg copper aspirinate for 7 days,significantly prolonged bleeding time and inhibited AA−induced mortality. The bleeding time of copper aspirinate−treated group was prolonged to 17.8 min from 11.6 min for the control group but aspirin showed only a non−significant tendency to prolong bleeding time at a dose of 0.056mmol/kg. This result is consistent with the known observation that aspirin lacks the ability to prolong bleeding time although it is a typical antiplatelet drug. Mortality of mice was decreased from 87% for the control group to 13% by copper aspirinate but to 27% by aspirin. Bleeding time of damaged blood vessels is closely related to the thrombosis at damaged sites. It is,therefore,obvious that copper aspirinate possesses a much higher antithrombotic activity than aspirin Data presented in Table 3 show that the group of gerbils treated with copper aspirinate prior to ischemia had a greater survival than the vehicle−treated group,while there was no significant difference in mortality between groups of copper aspirinate and nimodipine,mortality being 7% and 6%,respectively. From changes in the brain calcium content during ischemia and reperfusion (see Table 4),copper aspirinate was found to be very effective in lowering the brain calcium concentration of gerbils,exhibiting an anti−overloaded calcium effect. This effect of copper aspirinate at 0.018mmol/kg ig seems to be greater than that of nimodipine at 0.024mmol/kg ip. However no significant differences in brain water content were observed among these groups. As shown in Table 5, 0.018mmol/kg ig copper aspirinate,similar to 0.024mmol /kg ip nimodipine,markedly increased the neural cell density in the CA1 sector of the hippocampus of pretreated animals. This means that treatment of copper aspirinate could protect hippocampal CA1 cells from damage by ischemia.

Based on the decreased mortality,decreased calcium content and increased neural density we conclude that the anticerebral ischemia effects of copper aspirinate are comparable to that of nomidipine in spite of the difference in administration route between these two drugs in our experiments.

It has been shown in our previous studies [4] that copper aspirinate significantly increases $PGI_2$ in plasma while inhibiting synthesis of $TXA_2$,in constrast to aspirin which can only decrease the formation of $TXA_2$ and to nimodipine which acts as a vasodilator. $PGI_2$ is known to be a powerful vasodilator. So, copper aspirinate is expected to exhibit both effect of aspirin as an antiplatelet drug and of nimodipine as a vasodilator. Therefore copper aspirinate will become a more effective drug in preventing and treating thrombotic diseases, particularly cerebral ischemia companied by abnormalities of platelet aggregation.

**References**

1. J.R.J.Sorenson,*Prog.Med.Chem.*,1989,26,437
2. Liu Weiping, Li ling, *Academic J.Kunming Med.College*,1996,17(3),1
3. L.J.Hayden,C.Thomas and G.B.West,O,*J.Pharm.Pharmacol.*,1978,30,244
4. Shen Zhiqiang,Chen Zhihe,Liu Weiping,*Acta Pharmacologica Sinica*,1997,18(3), 358
5. J.R.J.Sorenson,*J.Med.Chem.*,1976,19(1),135
6. J.P.Wang,M.F.Shu,*Throm.Res.*,1985,37,669
7. C.Kohler,W.Wooding,L.Ellenbogen,*Throm.Res.*,1976,19,67
8. W.Youny, L.Vajda, K.A.Hossman, *Shock*,1987,18,751
9. E.N.Peterson, *Pharmacol.Toxicol.*,1989,65,299

**Received: December 12, 1997 - Accepted: January 6, 1998 - Received in revised camera-ready format: March 18, 1998**

